# Supplementary material for: Glycoproteomic Analysis of Urinary Extracellular Vesicles for Biomarkers of Hepatocellular Carcinoma
Source: Molecules. 2023 Jan 29;28(3):1293. doi: 10.3390/molecules28031293 (PMC9919939; doi:10.3390/molecules28031293)
Supplement: Supplementary file 1 [file molecules-28-01293-s001.zip › molecules-2153141-supplementary-Figure and Table.pdf]

Supplementary Materials for

Glycoproteomic Analysis of Urinary Extracellular Vesicles for Biomarkers of Hepatocellular Carcinoma

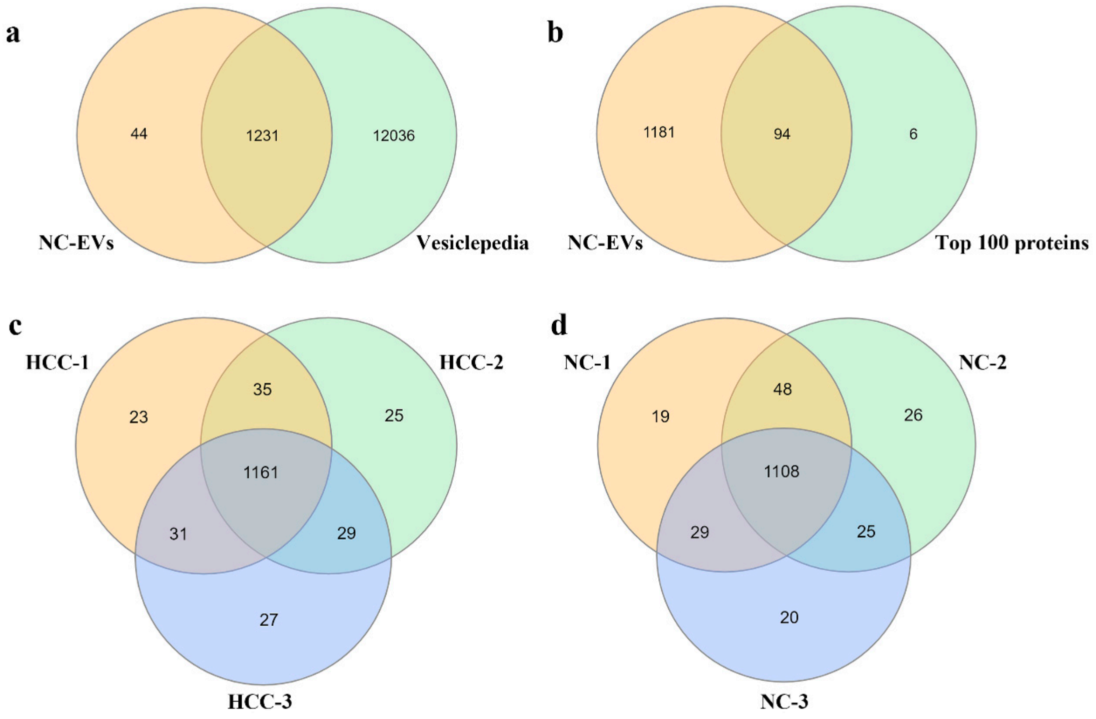

Figure S1. Evaluation of the urinary EVs isolated by EVTRAP method derived from normal controls (NC). (a) The Venn diagram depicted the overlap of proteins identified in urinary EVs derived from NC with those published in the Vesiclepedia database; (b) Overlapping with the top 100 proteins in the Vesiclepedia database; (c, d) Overlap with three needles in the proteomic analysis of urinary EVs from HCC and NC, respectively.

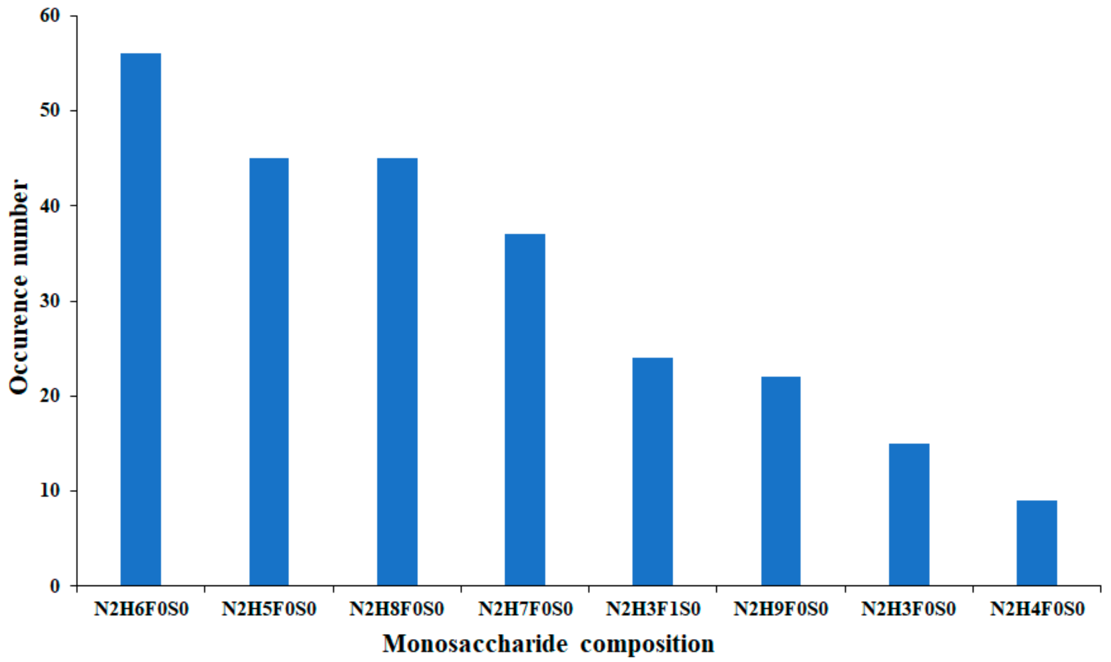

Figure S2. The eight most common N-glycan monosaccharide compositions vs the occurrence number of intact N-glycopeptide IDs identified in this study.

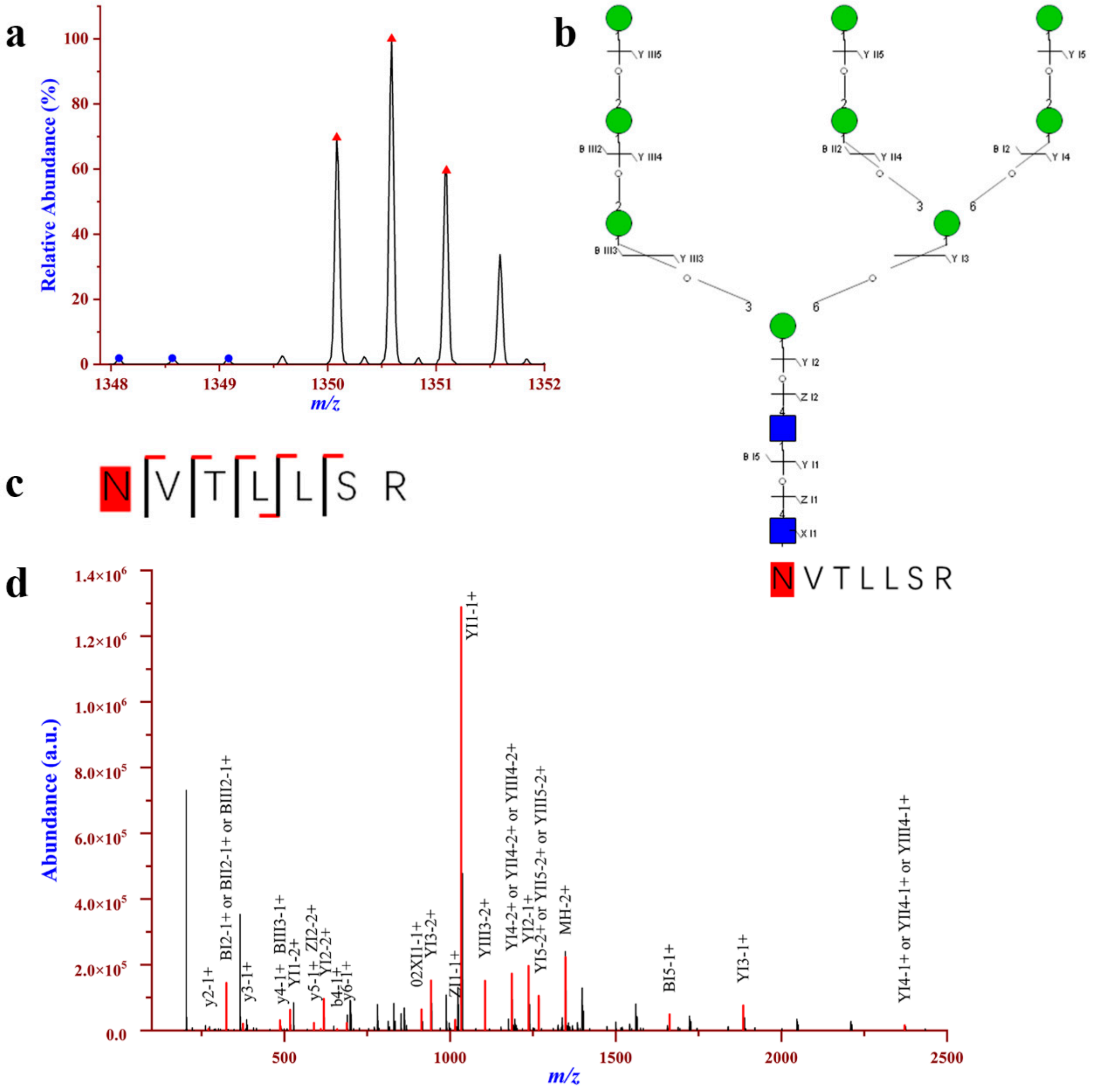

Figure S3. Upregulation of intact N-glycopeptide NVTLLSR_N2H9F0S0 in urinary EVs from patients with HCC relative to NC; the N-glycosite is N828 on Glutamyl aminopeptidase (AMPE_HUMAN, Q07075). (a) paired precursor ions. (b, c) N-glycan and peptide backbone graphical fragmentation maps annotated with the matched fragment ions. (d) The MS/MS spectrum with the matched fragment ions.

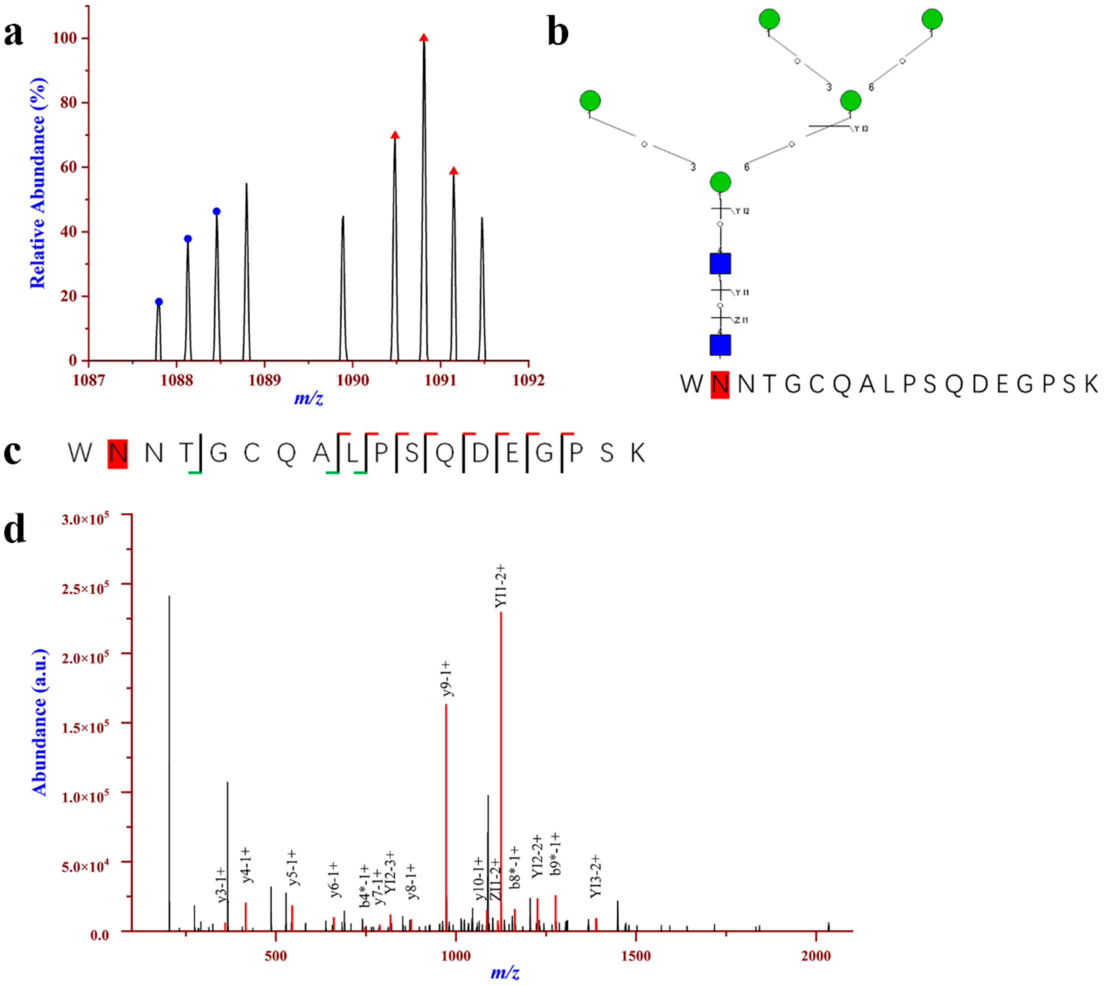

Figure S4. Upregulation of intact N-glycopeptide WNNTGCQALPSQDEGPSK_N2H5F0S0 in urinary EVs from patients with HCC relative to NC; the N-glycosite is N499 on Polymeric immunoglobulin receptor (PIGR_HUMAN, P01833). (a) paired precursor ions. (b, c) N-glycan and peptide backbone graphical fragmentation maps annotated with the matched fragment ions. (d) The MS/MS spectrum with the matched fragment ions.

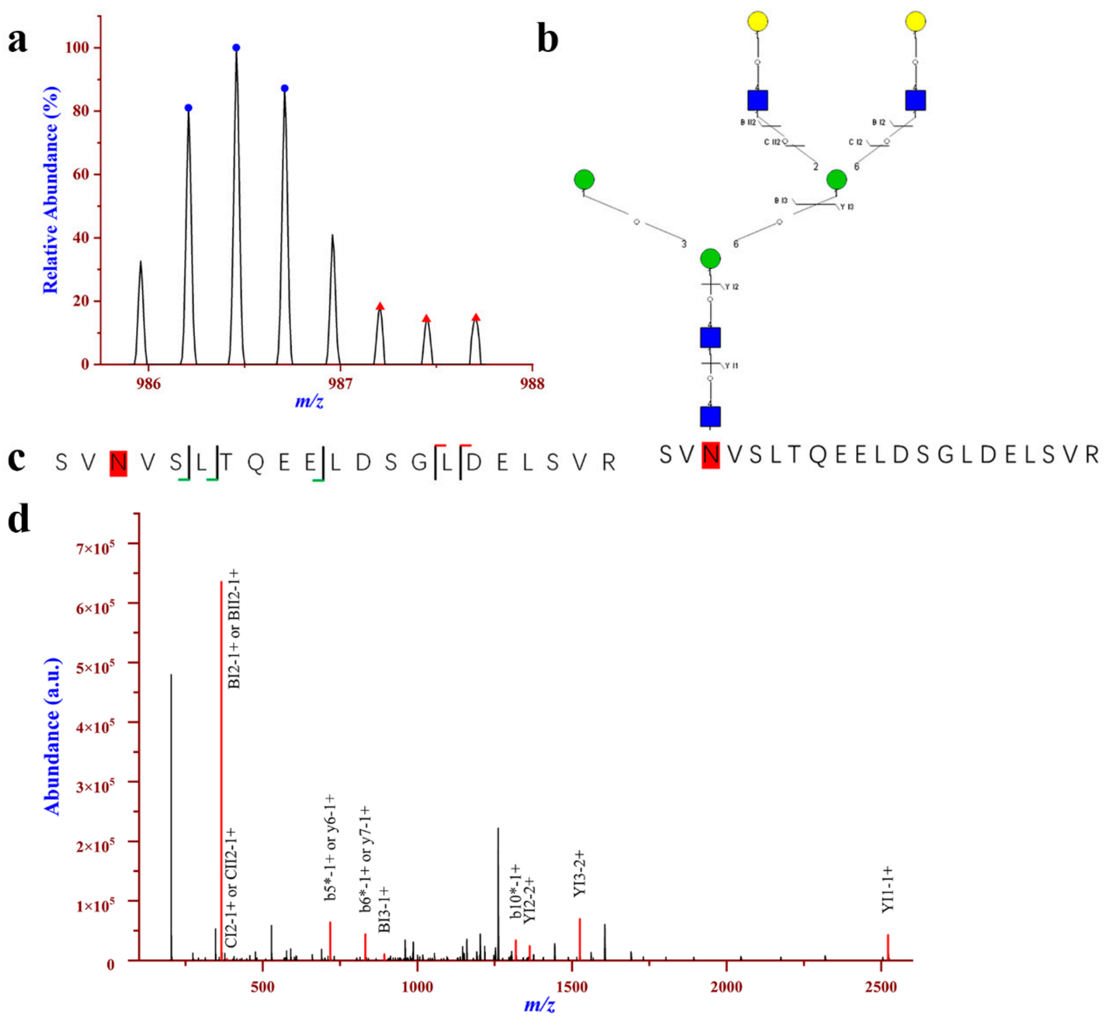

Figure S5. Downregulation of intact N-glycopeptide SVNVSLTQEELDSGLDELSVR_N4H5F0S0 in urinary EVs from patients with HCC relative to NC; the N-glycosite is N463 on A-kinase anchor protein 2 (AKAP2_HUMAN, Q9Y2D5). (a) paired precursor ions. (b, c) N-glycan and peptide backbone graphical fragmentation maps annotated with the matched fragment ions. (d) The MS/MS spectrum with the matched fragment ions.

Table S1. Clinical characteristics of the HCC patient enrolled in this study.

No.	Gender	Age	Liver cirrhosis [a]	HBsAg (Hepatitis B surface antigen) [b]	HBcAb (Hepatitis B core antibody) [b]	HCV antibody [b]	NASH [b]	Tumor number	Size of tumor (cm) [c]	Lymphatic metastasis [a]	AFP (ng/L) [c]	PIVKA Ⅱ (ng/L) [c]	Child-Pugh Score	Differentiated degree [c]
HCC1	male	64	1	N	N	P	0	3	2.1×1.7；2.2×1.8；1.6×1.4	1	804.34	642.56	B	Ⅲb/C
HCC2	female	75	1	N	N	N	1	＞3	the largest,3.7×2.9	1	400.93	3256.75	C	Ⅳ/D
HCC3	female	65	0	P	P	N	0	2	5.0×4.7；1.9×1.7	1	306.65	87.34	A	Ⅲb/C
HCC4	male	55	1	N	P	N	0	1	4.8×6.7	1	407.07	36.12	B	Ⅲb/C
HCC5	male	65	1	N	N	N	0	2	2.9×2.6；3.2×2.7	0	8.23	N/A	A	Ⅱa/B
HCC6	female	58	1	N	N	P	0	2	2.4×1.8；1.9×2.5	0	3.31	N/A	A	Ⅰb/A
HCC7	male	62	1	N	N	P	0	1	3.9×3.4	1	2.24	1829.91	B	Ⅲb/C
HCC8	female	64	1	N	N	N	0	＞3	the largest,1.9×1.5	1	468	383.42	A	Ⅲb/C

HCC9	male	52	0	P	P	N	0	1	5.3×3.4	0	＞ 3000	N/A	B	Ⅲa/C
HCC10	male	54	1	P	P	N	0	2	4.8×5.6;3.9×2.7	1	14.81	826.33	A	Ⅲb/C
HCC11	male	54	0	P	P	N	0	1	2×2.3	0	2.99	N/A	A	Ⅰa/A
HCC12	male	53	1	N	P	N	0	2	2.6×1.9；3.5×3.8	1	752	743.26	C	Ⅳ/D
HCC13	female	70	1	P	P	N	0	1	3.2×3.3	0	2823.15	66.57	B	Ⅰa/A
HCC14	male	53	1	N	P	N	0	1	3.5×3.4	1	652.06	339.42	B	Ⅲb/C
HCC15	female	38	0	P	P	N	0	1	1.8×1.5	0	387.42	66.88	A	Ⅰa/0
HCC16	female	57	1	N	N	P	0	2	3.3×3.5；2.2×2.5	0	10.58	N/A	A	Ⅱa/B
HCC17	male	44	0	N	N	P	0	＞3	the largest,5.2×5.5	0	201.61	468.34	A	Ⅱb/B
HCC18	female	69	1	N	N	N	1	1	11.4×10..8	1	1872	N/A	C	Ⅳ/D
HCC19	male	55	1	N	N	N	0	＞3	the largest,3.5×2.9	0	976.42	455.21	A	Ⅱb/B
HCC20	male	34	0	P	P	N	0	1	5.5×4.8；	0	＞ 3000	23.56	A	Ⅰb/A
HCC21	female	48	1	P	P	N	0	2	6.7×5.7;3.3×3.7	0	2668.45	7.67	B	Ⅲa/C
